# Spatial Immunology in Translation: Linking Immune Organisation to Therapeutic Outcome

**DOI:** 10.3390/medsci14020308

**Published:** 2026-06-12

**Authors:** Caio Santos Bonilha

**Affiliations:** Institute of Infection, Immunity and Inflammation, University of Glasgow, Glasgow G12 8TA, UK; caio.bonilha@glasgow.ac.uk

**Keywords:** spatial transcriptomics, translational immunology, inflammation, immune microenvironment, tissue architecture

## Abstract

Immune-targeted therapies are commonly interpreted through molecular and cell-centric frameworks that insufficiently capture how immune activity is organised within intact tissues. This limitation complicates translational interpretation of therapeutic efficacy and response variability when inflammatory activity is spatially structured within diseased tissue. This review examines immune organisation as a relevant dimension of immune function and frames interaction-defined immune environments as functional units of inflammation. It outlines how cellular composition, tissue compartmentalisation, and persistence of interaction environments shape where immune signalling is concentrated and sustained in situ. By linking immune organisation to tissue-level behaviour, the review provides translational context for interpreting target engagement and therapeutic effects, supporting more informed alignment between therapeutic strategies and the immune architectures that dominate disease activity.

## 1. Spatial Context as an Underweighted Dimension in Immune Therapies

Spatial organisation has long been recognised as a fundamental feature of immune function, shaping how immune cells encounter antigens, establish contacts, and integrate signals within tissues. Concepts such as compartmentalisation, tissue residency, and microenvironmental conditioning have been central to immunological thinking and to the interpretation of immune responses across organs [1,2]. Experimental and pathological observations consistently demonstrate that immune activity is constrained by physical proximity, local mediator availability, and tissue architecture [3,4,5,6,7,8,9]. Despite this well-established biological understanding, spatial organisation has most often been incorporated as contextual background that supports mechanistic insight, with more limited influence on how therapeutic strategies are formulated [10]. This tendency is apparent in the development of immune-targeted therapies, which have commonly relied on molecular- and cell-type-focused evidence derived from bulk tissue or circulating immune analyses. Such compression of spatially organised immune biology into averaged signals obscures where pathogenic immune activity is concentrated and contributes to variable efficacy, unexpected toxicity, or discordant biomarker responses across diseases.

Recent advances in spatially resolved analysis have reshaped how immune activity can be examined within intact tissue environments. Approaches such as spatial transcriptomics and multiplex imaging enable immune features to be analysed in relation to tissue architecture and local cellular organisation, providing insight into where immune pathways are engaged within diseased tissue [11,12]. These developments allow spatial information to function as an analytical dimension that informs interpretation of immune activity within tissues. In this review, spatial immunology is considered as a conceptual framework for understanding immune mechanisms through interaction context and tissue organisation, and for evaluating therapeutic targets according to the settings in which they exert pathogenic effects. By focusing on organised immune interactions embedded within tissues, this perspective reframes translational reasoning around the functional contexts that ultimately shape therapeutic outcome.

## 2. Immune Interaction Niches as Functional Units of Inflammation

### 2.1. From Cell Types to Interaction-Defined Niches

**Figure 1:**
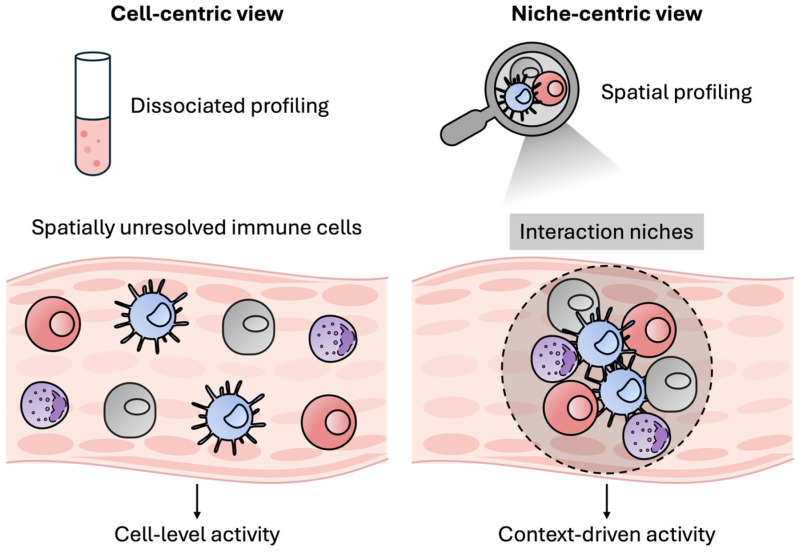
Cell-centric versus niche-centric views of immune activity within tissue. Conceptual comparison between cell-centric and niche-centric interpretations of immune organisation. In the cell-centric view, immune activity is interpreted through the presence, abundance, and activation state of individual cell types, typically inferred from bulk or dissociated profiling approaches that average spatial information. In the niche-centric view, immune function emerges from spatially organised interaction niches, where coordinated cell–cell contacts and local signalling environments shape inflammatory or regulatory outputs. Distinct niches within the same tissue can support divergent functional programmes, highlighting how spatial context integrates immune signals beyond cell identity alone.

Inflammation is often first interpreted through a cell-type lens, where immune behaviour is attributed to lineage identity and cell-intrinsic programmes. This approach has strong mechanistic roots and remains central to how pathways are mapped to discrete populations, how biomarkers are interpreted, and how targets are prioritised. Bulk tissue profiling and dissociated single-cell readouts reinforce this logic by emphasising transcriptional states, receptor expression, and pathway activation within defined cell subsets. Within this framework, the tissue is frequently treated as a container in which activated cell types accumulate, and disease is explained through the presence, abundance, or activation of those populations. This cell-type emphasis remains highly informative, yet it places the burden of explanation on intrinsic cellular properties, even when tissue biology suggests that context and coordination play a decisive role (Figure 1).

Tissue-level observations introduce persistent tension with a purely cell-centric interpretation. The same immune cell types can display distinct functional features depending on local positioning, neighbouring populations, and the surrounding tissue state, even within the same biopsy. In inflamed skin [13,14,15], synovium [16,17], and mucosal tissues [18,19,20,21], pathogenic activity often concentrates in spatially discrete regions, while adjacent areas containing similar lineages exhibit muted activation and different effector outputs. A related principle is evident in solid tumours, where tertiary lymphoid structure-rich regions can support organised antigen presentation, B cell maturation, and T cell activation, while immune-excluded tumour–stromal interfaces can restrict effector access and maintain local immune dysfunction despite the presence of immune cells within the broader lesion [22,23,24]. This spatial clustering is repeatedly accompanied by coordinated transcriptional signatures across multiple immune populations, consistent with shared local drivers and reciprocal reinforcement. Such patterns can be diluted when tissue is homogenised and can be difficult to reconstruct from cell-intrinsic profiles alone, particularly when key signals depend on proximity, contact, and local availability of mediators. These features push interpretation toward a framework that treats coordinated cellular assemblies as the unit of inflammatory activity.

These spatially clustered patterns of immune activity point toward a role for proximity, contact, and local signalling environments to shape how immune programmes are expressed within tissue. Close spatial arrangement permits repeated cell–cell engagement and spatially reinforced signal integration that are unlikely to occur uniformly across an organ, instead emerging within confined cellular neighbourhoods. At the same time, neighbouring regions that contain similar immune lineages can experience different signalling pressures depending on local tissue state and cellular composition [25]. Such observations suggest that immune behaviour is organised within constrained interaction settings that influence activation, coordination, and persistence of inflammatory responses. However, the properties that distinguish one interaction setting from another, and that determine whether these settings support transient immune activity or sustained inflammation, remain variable across tissues and disease contexts [25,26]. Addressing this variability requires closer examination of the factors that govern how interaction-defined environments are structured and maintained.

### 2.2. Determinants of Niche Functionality

One of the most immediate factors shaping the behaviour of interaction-defined environments is the cellular composition within a given spatial setting. The relative abundance of immune populations influences the balance of signals that can be generated and sustained locally. Small changes in the proportion of myeloid, lymphoid, or stromal cells can shift dominant signalling axes and alter the coordination of immune programmes [27,28]. Within tissues, enrichment of specific cell combinations often coincides with amplification of effector pathways, while neighbouring regions with similar cell types but different proportions display attenuated responses [29,30]. These patterns highlight that niche output reflects collective cellular presence and density, not simple coexistence. In tumour immunity, this principle is illustrated by ILC2s and tertiary lymphoid structures, where IL-33-responsive ILC2s can promote lymphotoxin-dependent TLS formation in pancreatic cancer [31], and ILC2 activity has also been linked to eosinophil recruitment [32], immune checkpoint responsiveness, and anti-tumour regulation in melanoma [33]. As immune cells accumulate or recede in response to inflammatory cues, the functional character of an interaction setting can evolve, setting the stage for additional layers of regulation that stabilise or modulate these assemblies. Representative examples of immune interaction niches identified across tissues and disease contexts are summarised in Table 1, spanning interaction environments that evolve with inflammatory state as well as niches stabilised by tissue architecture.

Beyond cellular composition, the stability and specificity of immune interaction settings depend on molecular systems that support sustained cell–cell engagement. Adhesion molecules and co-signalling pathways shape stable immunological contacts that consolidate signalling networks and coordinated activation across neighbouring cells [36,37]. Their expression and activation often increase within inflamed tissue, promoting prolonged interactions that extend beyond transient encounters. These molecular interfaces influence how signals are integrated across participating cells and how local activation thresholds are maintained. Variation in adhesion and co-signalling availability can therefore modulate the intensity and duration of immune activity within a given setting. Importantly, these systems operate within the spatial constraints imposed by tissue structure, linking molecular engagement to physical context and preparing the ground for architectural influences on niche behaviour.

Tissue architecture provides a structural framework that constrains where and how immune interaction settings can form. Barriers, vascular networks, epithelial layers, and stromal compartments actively organise immune cell access and retention, shaping which cells can interact and the duration of those interactions within defined tissue ecosystems [34,35,38,39]. Lymphatic vessels add a further organising layer, with lymphatic remodelling shaping tumour immune microenvironments [40], lymphangiogenesis blockade altering macrophage-rich tumour-associated inflammation [41], and tumour lymphangiogenesis enhancing T cell infiltration and immunotherapy responsiveness in melanoma [42]. These architectural features can focus immune activity into spatially restricted regions or partition interactions across microanatomical boundaries that impose functional separation [43]. Within inflamed tissue, remodelling of these structures can further alter interaction landscapes by modifying cell positioning and local connectivity. Such changes may stabilise immune activity within specific regions or enable its spread into adjacent compartments. As tissue structure evolves during inflammation, it interacts with cellular and molecular determinants to shape the persistence and distribution of immune interaction environments.

The functional properties of immune interaction settings also vary over time as inflammatory processes progress. Early inflammatory phases often involve transient assemblies that support rapid activation and signal exchange, while prolonged inflammation favours more stable configurations with reinforced cellular contacts and sustained signalling [44,45,46]. Disease stage influences cellular composition, molecular engagement, and tissue architecture simultaneously, leading to shifts in how interaction environments operate. These temporal dynamics contribute to heterogeneity both within and across tissues, as similar interaction settings can support different outcomes depending on inflammatory context. Recognition of this variability underscores that immune interaction environments are not static entities but evolving features of tissue inflammation, a concept that becomes particularly relevant when considering how immune modulation engages dynamic inflammatory systems over the course of disease.

## 3. Why Molecular Targeting Alone Might Not Predict Therapeutic Outcome

**Figure 2:**
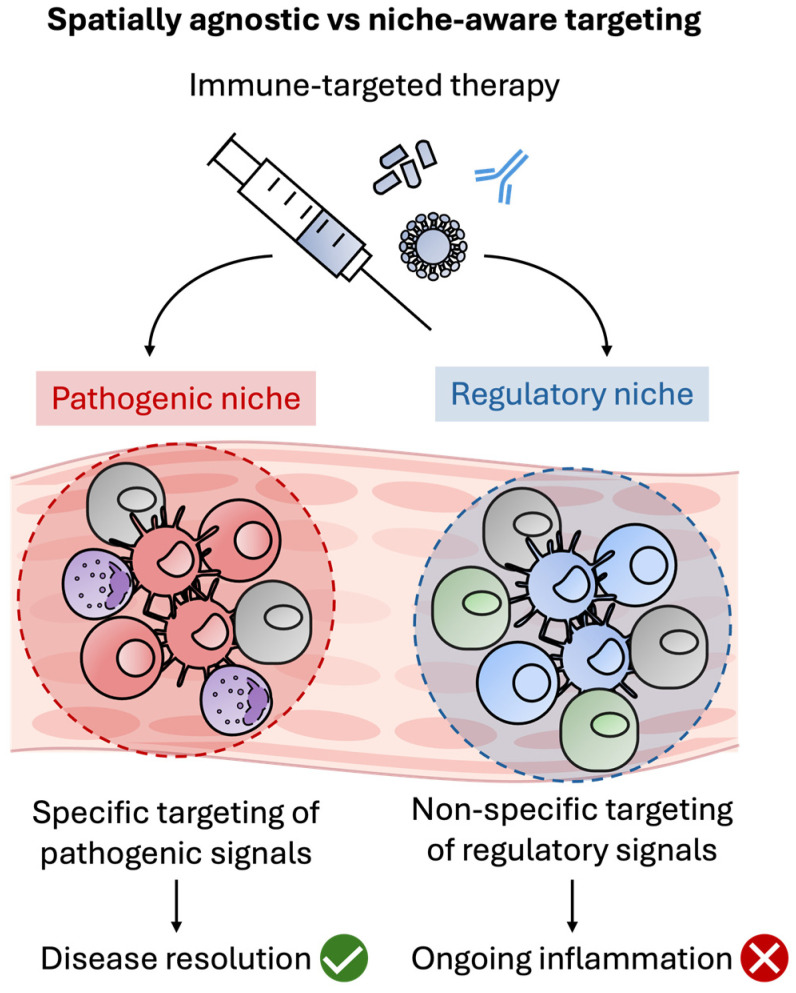
Spatial organisation shapes therapeutic outcome following molecular targeting. Schematic illustrating how identical molecular intervention can produce divergent tissue-level outcomes depending on the spatial organisation of immune interaction niches. A single tissue contains coexisting pathogenic and regulatory interaction environments with distinct cellular composition and signalling roles. Systemic pathway modulation engages signalling across these spatially segregated niches simultaneously. As a result, biological outcomes reflect how therapeutic effects are distributed across interaction environments with different functional roles within tissue. The figure emphasises that tissue-level response is shaped by spatial organisation of immune activity, not solely by the presence or inhibition of a molecular target. The green tick indicates the desired therapeutic outcome, whereas the red cross indicates an unfavourable inflammatory outcome.

Variability in efficacy, safety, and tissue responsiveness is a common feature of immune-targeted interventions across inflammatory diseases. Agents that effectively engage their intended targets can nonetheless produce heterogeneous biological and clinical outcomes across tissues, disease stages, or patient groups [47,48,49,50]. These patterns are often attributed to disease complexity, pathway redundancy, or differences in inflammatory burden. A complementary consideration is how immune signalling is organised within tissues, where pathological activity is often concentrated in specific interaction settings. When signalling relevance is unevenly distributed across tissue regions, modulation of a pathway can have disproportionate biological consequences depending on which interaction settings dominate disease activity at a given time [16,47,48,51,52] (Figure 2). Incorporating this dimension into interpretation helps reconcile why similar degrees of target engagement can be associated with divergent outcomes, without invoking failure of inhibition or intrinsic unpredictability of immune responses.

### 3.1. Spatial Context and Variability in Immune Signalling Outcomes

Within inflamed tissues, engagement of identical ligand–receptor pairs can give rise to distinct biological outcomes depending on the spatial setting in which signalling occurs. The same molecular interaction may support effector activation in one region while contributing to regulatory or reparative processes in another, even within the same tissue [53,54]. Such divergence reflects differences in local cellular composition, signal intensity, and the duration and frequency of cell–cell contact. Spatial proximity shapes receptor clustering and downstream signal integration, influencing transcriptional and functional responses without changes in molecular identity. Tissue-resolved analyses consistently show that pathway activity aligns with local interaction environments defined by coordinated cellular organisation and tissue context [20,21,25,26,27,28,30,35,38,43,48,51,55,56,57]. This spatial dependence becomes particularly relevant when signalling pathways are modulated across entire tissues through therapeutic intervention.

Broad modulation of immune pathways acts simultaneously on signalling occurring within multiple spatial interaction environments distributed across tissue. Some of these environments contribute directly to disease activity, while others participate in regulation or tissue maintenance, with distinct spatial immune architectures linking local organisation to differential therapeutic outcomes [47,52,58]. For pathways that depend on contact-dependent or locally amplified signalling, intervention suppresses activity across these distinct settings at the same time. The biological readout that follows therefore reflects combined effects arising from interaction environments with unequal relevance to pathology. This blending of spatially distinct contributions can complicate interpretation of tissue-level responses, especially when pathogenic signalling represents only a fraction of the contexts in which a pathway is active. As a result, the relationship between target engagement and observable outcome becomes less straightforward.

Variation in therapeutic response can emerge from differences in how spatial interaction environments are distributed and maintained across tissues and disease stages. Shifts in cellular organisation, local tissue structure, or inflammatory state can alter which interaction settings exert the strongest influence on overall immune activity. When signalling effects are expressed within confined spatial environments, even modest differences in tissue organisation can change how intervention manifests at the biological level [59]. Similar degrees of pathway inhibition may therefore be associated with distinct outcomes depending on which interaction settings dominate disease activity in each context. This view highlights how broad intervention can influence pathogenic and beneficial interactions in parallel—an issue that becomes increasingly salient when considering how spatial specificity is lost during immune modulation.

### 3.2. Off-Target Effects Arising from Spatially Unresolved Intervention

Therapeutic modulation is typically delivered at a scale that exceeds the spatial confinement of immune interaction environments within tissue [60]. Systemic or tissue-wide inhibition therefore acts on signalling processes that are active across multiple spatial settings simultaneously, including those that differ in cellular composition, functional role, and contribution to pathology within organised tissue microenvironments [47,58]. As spatially organised signalling can support both pathogenic and tissue-supportive immune programmes within the same tissue, inhibition propagates across settings with unequal relevance to inflammatory burden. The resulting biological response may therefore reflect the combined disruption of spatially distinct signalling contexts rather than selective modulation of pathogenic activity. This spatial decoupling between drug exposure and functional relevance introduces a layer of collateral immune perturbation that is independent of molecular specificity. As intervention proceeds, immune activity is reshaped across diverse interaction environments, setting the stage for effects that extend beyond the processes sustaining disease activity.

The consequences of this spatially uncoupled modulation become evident at the level of tissue behaviour, extending beyond what can be anticipated from target identity alone. Interaction environments involved in regulation, repair, or barrier support can be altered alongside those associated with inflammation, leading to shifts in local immune coordination that affect epithelial integrity and immune-mediated tissue balance [61,62]. These effects may manifest as altered tissue resilience, delayed resolution, or compensatory activation of parallel pathways that restore local signalling capacity. Such outcomes may reflect disruption of spatial organisation without implying incomplete target engagement or pathway redundancy as the primary explanation. In this context, the immune system can adjust to perturbation by redistributing activity across remaining interaction environments, enabling functional outputs to persist even when a targeted pathway is broadly suppressed [63,64]. This redistribution contributes to variability in therapeutic readouts and can obscure the relationship between inhibition and disease control.

Over time, the cumulative impact of spatially decoupled inhibition influences how immune activity is maintained and perceived at the tissue level. Measurements derived from bulk tissue or systemic sampling capture net changes in signalling without resolving where those changes occur or which interaction environments are most affected [10,11,47,60,63]. Apparent improvement may therefore coexist with ongoing pathogenic coordination within confined regions, while adverse effects may reflect disruption of interaction settings that support regulation or tissue maintenance. This mismatch between spatial organisation and intervention scale complicates interpretation of efficacy and safety across diseases and patient groups. Recognition of this limitation creates a conceptual bridge toward strategies that align therapeutic action with the spatial logic of immune activity, providing continuity with subsequent consideration of niche-aware design.

### 3.3. Spatial and Temporal Alignment of Immune Modulation

Therapeutic outcome can depend on whether immune modulation is aligned with the spatial and temporal settings in which disease-relevant activity is organised. In sepsis, inflammatory and immunosuppressed states can differ across patients and evolve over time, allowing the functional relevance of the same immune pathway to shift according to disease stage and dominant immune state. Recent precision immunotherapy approaches reflect this principle by assigning treatment according to hyperinflammatory or immunoparalysed immune states, including targeted blockade of IL-1-driven inflammation, supporting context-matched intervention as a strategy for improving therapeutic interpretation and clinical effect [65]. A related logic applies in tumour immunotherapy, where intratumoural activation of innate immune sensing combined with checkpoint blockade can focus therapeutic activity within the lesion and engage organised suppressive and effector environments at the site of disease [66]. These settings show how spatial and temporal awareness can refine interpretation of therapeutic response and support closer alignment between intervention and disease-relevant immune organisation.

## 4. Toward Niche-Aware Therapeutic Design

**Figure 3:**
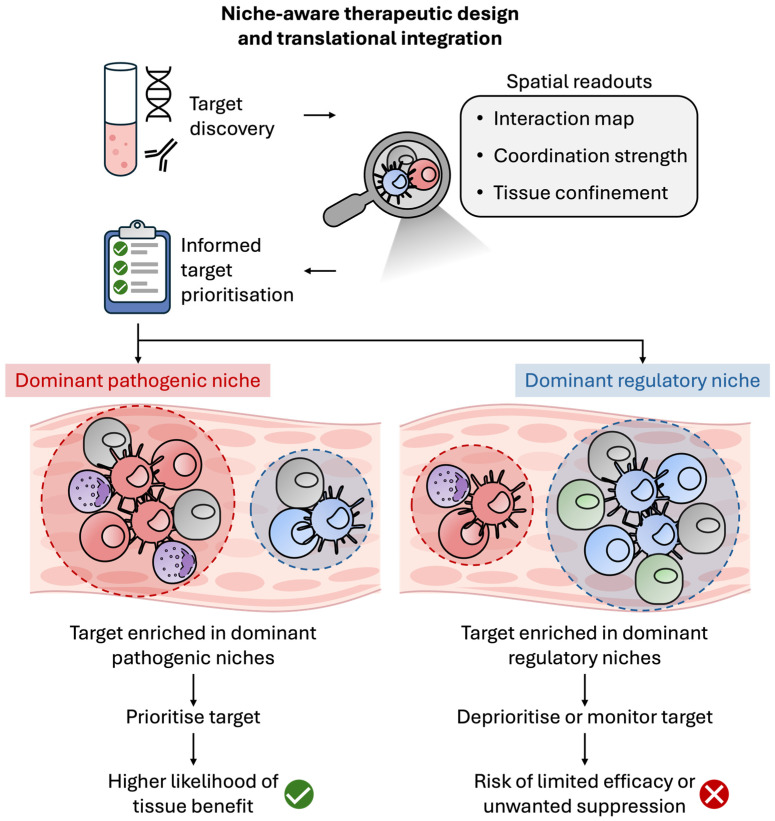
Spatially informed framework for niche-aware therapeutic prioritisation. Conceptual framework depicting how spatial immunology informs translational target evaluation. Spatial readouts such as interaction maps, coordination strength, and tissue confinement provide information on where immune signalling is organised within tissue. Distinct tissue contexts can contain both pathogenic and regulatory niches, with dominance indicated by relative cellular abundance and interaction density. Spatial information allows assessment of whether a candidate target is preferentially engaged within the dominant pathogenic niche or within regulatory interaction environments. Targets aligned with dominant pathogenic niches are prioritised for intervention and associated with resolution of tissue inflammation, whereas targets predominantly engaged within regulatory niches are deprioritised due to limited therapeutic leverage or potential disruption of immune regulation. This framework positions spatial organisation as an informative layer guiding translational decision making. The green tick indicates a favourable prioritisation outcome, whereas the red cross indicates a target profile associated with limited efficacy or unwanted immune suppression.

The challenges posed by spatially unresolved immune modulation motivate a shift in how therapeutic strategies are conceived and evaluated. When immune activity is organised within discrete interaction environments, aligning intervention with the contexts that sustain pathology becomes essential for achieving meaningful efficacy while limiting collateral impact. Niche-aware therapeutic design addresses this misalignment by situating target selection within the spatial organisation of immune activity itself. In this framework, interaction environments provide contextual information that complements molecular and pharmacological criteria, creating a basis for evaluating therapeutic relevance that reflects how immune mechanisms operate within tissue (Figure 3).

### 4.1. Spatial Features That Define Actionable Targets

Once interaction environments are considered as a layer of therapeutic context, spatial features become informative for target prioritisation. Interaction density, co-localisation of immune populations, and confinement to defined tissue compartments indicate where signalling is concentrated and coordinated. Targets enriched within regions of dense immune interaction are positioned within collective activity that drives local pathology [16,30,67,68]. Spatial restriction further differentiates targets linked to disease-associated regions from those broadly distributed across tissue. These features provide a means to assess relevance that extends beyond expression level alone, and begin to distinguish targets according to their association with niche-level immune organisation. Key spatial features that refine therapeutic target selection are summarised in Table 2.

Spatially resolved analyses can strengthen therapeutic target evaluation by linking candidate pathways to the tissue settings in which immune activity is organised. This is particularly relevant in tumour immunity, where the same lesion can contain immune-activating regions, exclusion zones, and stromal compartments with different implications for therapeutic response. In nasopharyngeal carcinoma, spatial coupling between tertiary lymphoid structures and tumour-cell aggregates identified regions where organised antigen presentation and lymphocyte activation were linked to immunotherapy-relevant immune architecture [23]. This type of organisation contrasts with settings in which immune activity is present but spatially separated from effective tumour control, as shown in lung cancer with mature tertiary lymphoid structures, where cancer-associated fibroblast-rich regions were associated with T cell dysfunction and resistance to checkpoint blockade [69]. Spatial evaluation can also broaden target selection beyond immune-cell positioning by identifying therapeutic opportunities within the tumour stroma. In immunotherapy-exposed clear cell renal cell carcinoma, spatial coupling between integrin and collagen programmes highlighted compartment-specific cell–matrix interactions as candidate targets [70]. These applications show that spatial features refine target selection most effectively when they identify candidate pathways within the tissue contexts that shape therapeutic response.

Beyond their spatial localisation, interaction environments differ in stability and cellular specificity, which further shape how targets are evaluated. Some environments persist across disease stages, while others appear transiently in response to fluctuating inflammatory cues [26,71]. Targets associated with persistent environments may exert influence across broader phases of disease, whereas those linked to transient settings may reflect stage-restricted processes. Cellular specificity also varies, as signalling confined to narrowly defined interaction environments differs from signalling distributed across multiple contexts [48,72,73]. Consideration of these attributes refines prioritisation by linking target activity to interaction environments that are both spatially focused and functionally coherent.

### 4.2. Integrating Spatial Readouts into Translational Pipelines

Translation of niche-aware concepts into therapeutic development requires points of contact between spatial information and established discovery workflows. Spatial transcriptomics and multiplex imaging provide tissue-resolved views that can contextualise targets emerging from genetic association, perturbation screens, or functional assays [10,11,74]. Their value lies in clarifying whether candidate targets are engaged within interaction environments that concentrate disease-relevant signalling. Applied at defined stages, spatial readouts can inform decisions about target prioritisation, mechanism plausibility, and tissue relevance without displacing upstream discovery approaches. In this capacity, spatial data operate as an interpretative layer that links molecular findings to the organisation of immune activity within tissue.

At later translational stages, spatial information can refine alignment between therapeutic mechanism and patient heterogeneity. Variation in clinical response can be interpreted through differences in how immune interaction environments are distributed across tissues and disease stages. Spatial profiling enables assessment of whether a target is active within the interaction settings that dominate a given tissue context in a subset of patients. When combined with molecular and clinical data, this information supports interpretation of response variability through tissue organisation rather than target presence alone. Integration at this level allows spatial context to inform stratification strategies that remain grounded in biological mechanisms.

Clinical implementation of niche-aware design is likely to depend on spatial readouts that can be reduced to reproducible, pathology-compatible measurements that retain mechanistic meaning. Translational use may emerge through focused measurements of immune localisation, stromal restriction, tissue compartmentalisation, and proximity between therapeutically relevant cell populations. Recent studies support this direction by showing that spatial organisation of immune and stromal states can improve interpretation of immunotherapy response [75], that digital histopathology can infer tumour microenvironment features linked to treatment outcome from routine tissue images [76], and that single-cell spatial profiling can connect clinical response with tissue-level cellular ecosystems [77]. In this form, niche-aware stratification becomes most feasible when spatial features are translated into standardised tissue measurements that complement established molecular and clinical biomarkers.

Computational frameworks are also becoming central to the translation of spatial readouts into interpretable immune features. Deconvolution and graph-based approaches can infer cellular composition and neighbourhood structure from spatial transcriptomic data, allowing candidate pathways to be mapped onto organised tissue regions [78]. Methods for spatial cell–cell communication inference further support identification of ligand–receptor activity within local interaction environments, linking molecular signalling to proximity and tissue context [79]. AI-driven analysis of histology and spatial datasets can extend these principles by integrating morphology, molecular state, and cellular organisation into scalable representations of tissue architecture [76]. These approaches can improve the reconstruction of immune interaction niches, although their outputs remain dependent on some practical limitations.

### 4.3. Practical Limitations and Strategies for Selective Implementation

Despite their conceptual and analytical value, spatial profiling approaches face practical constraints that shape their deployment within translational research. Spatial transcriptomics and high-parameter imaging require specialised infrastructure, remain resource-intensive, and support lower throughput than bulk molecular assays [80,81,82,83]. Limitations in sample availability, tissue quality, and analytical complexity further restrict scalability across large cohorts or early discovery stages. In addition, spatial assays involve inherent trade-offs between molecular breadth and tissue coverage, which can constrain comprehensive interrogation of immune pathways within a single experiment [82,83]. Important interpretative limitations also remain, including spot-level resolution in some transcriptomic platforms, incomplete capture of low-abundance mediators, uncertainty in cell segmentation or cell-type assignment, and tissue-processing effects that may alter local morphology or transcript detection [84,85]. These factors can influence how interaction environments are reconstructed and require spatial readouts to be interpreted alongside histological context, orthogonal molecular measurements, and functional validation. Recognition of these constraints supports selective implementation of spatial methods, with major technology classes and their translational applications summarised in Table 3 to guide method selection according to biological and clinical context.

A selective and question-driven application of spatial approaches provides a pragmatic response to these constraints. Spatial profiling has demonstrated value in settings marked by pronounced tissue heterogeneity, spatially restricted pathology, or misalignment between molecular target engagement and biological outcome—conditions that extend beyond any single disease category [82,83,86,87,88,89]. In such contexts, tissue organisation and interaction structure play a disproportionate role in shaping therapeutic response. Experimental modelling of these settings also requires careful choice of system. Animal models remain useful for testing tissue-level immune organisation in vivo, but differences in tissue architecture, stromal composition, immune development, and vascular or lymphatic structure can limit direct translation to human disease. Three-dimensional culture systems, including organoids and immune co-culture models, provide complementary platforms for dissecting defined cell–cell and cell–matrix interactions under controlled conditions, but they only partially reproduce the anatomical scale, vascularisation, innervation, and multicompartment organisation of intact tissue [90]. More broadly, spatial methods are most informative when deployed at decision points where tissue context resolves uncertainty around target relevance, mechanism, or response variability. Focused analysis of representative regions, guided by histological or molecular evidence, can preserve interpretative value while limiting resource demands. Complementary strategies, including targeted multiplex panels or region-aware expression analyses, further support incorporation of spatial reasoning across diverse disease settings while remaining aligned with practical and financial considerations.

## 5. Concluding Remarks and Future Directions

Immune responses within tissues emerge from organised patterns of cellular interaction that integrate molecular signalling with spatial context. Viewing inflammation through the lens of immune interaction niches provides a unifying framework that links spatial biology with therapeutic outcome, without displacing established principles of target engagement or pharmacology. This perspective brings coherence to observations of variable efficacy, safety, and tissue responsiveness by situating immune mechanisms within the interaction environments that sustain pathology. It also clarifies why modulation of the same pathway can yield distinct consequences depending on how immune activity is organised locally. Aligning mechanistic insight with tissue-level organisation offers a consistent way to interpret immune modulation across diseases and therapeutic classes, creating conceptual continuity between spatial immune biology and translational reasoning.

Future development of immune-targeted therapies will benefit from continued integration of spatial reasoning into experimental and translational frameworks. Advances in spatial profiling, imaging, and computational inference are expanding the capacity to interrogate immune organisation within intact tissues, while improvements in scalability and analytical efficiency are gradually lowering barriers to broader use. The impact of these approaches is greatest when applied selectively in settings where tissue organisation is a dominant determinant of disease behaviour or therapeutic response. As spatial information is integrated alongside genetic, functional, and clinical data, opportunities arise to align therapeutic strategies more closely with the interaction environments that shape immune pathology. Together, these directions point toward a translational landscape in which spatial organisation is treated as a core dimension of mechanism, interpretation, and design across immune-mediated disease.

## Figures and Tables

**Table 1:** Examples of immune interaction niches across tissues.

Niche Type	Dominant Cell Interactions	Tissue Context	Functional Outcome	Ref.
Dynamic interaction niches				
Metabolically coordinated immune niche	Immune cells engaging in inflammation-driven metabolic coordination within locally confined immune environments	Actively inflamed tissue regions	Sustained local amplification of type 17 immune programmes through dynamically regulated metabolic interactions	[14]
Neutrophil-centred antimicrobial niche	Spatially segregated neutrophil populations exhibiting distinct functional programmes within inflamed tissue	Inflamed mucosal tissue	Localised neutrophil effector activity with compartment-specific inflammatory and antimicrobial functions	[21]
Transient effector T cell interaction niche	Effector T cells engaging antigen-presenting cells within locally organised stromal interaction zones	Sites of acute immune activation	Coordinated signal exchange supporting T cell priming and early effector differentiation	[34]
Spatially anchored niches				
Fibroblast-organised immune niche	Activated fibroblasts coordinating immune cell positioning and signalling	Stromal tissue frameworks	Spatial confinement and reinforcement of inflammatory programmes	[18]
Vascular-associated immune niche	Perivascular macrophages organised around endothelial structures and interacting with infiltrating immune cells	Perivascular regions	Regulation of immune entry, positioning, and retention	[16]
Epithelial–immune interface niche	Epithelial cells engaging immune cells through contact and local mediators	Barrier tissues	Coupling of immune activation with tissue state	[35]
Resident memory immune niche	Tissue-resident memory T cells exhibiting region-specific differentiation and functional imprinting	Non-lymphoid tissues	Spatially imprinted immune surveillance with locally specialised functional programmes	[9]

**Table 2:** Spatial features that refine therapeutic target selection.

Spatial Feature	Description in Tissue Context	Translational Relevance for Target Selection
Niche restriction	Preferential localisation of target activity within discrete immune interaction environments	Prioritises targets engaged within spatially confined pathological settings
Cell–cell coupling strength	Dependence of signalling on sustained proximity or contact between specific cell populations	Identifies targets whose impact arises from coordinated interaction dynamics
Tissue compartment specificity	Enrichment of target activity within defined anatomical compartments	Distinguishes targets linked to disease-associated tissue regions
Spatial concordance with pathology	Alignment of target activity with regions of tissue damage or inflammatory remodelling	Supports prioritisation of targets relevant to active disease sites
Persistence across disease stages	Stability of target engagement within interaction environments over time	Differentiates targets linked to sustained pathology from transient responses
Spatial separation from regulatory niches	Exclusion of target activity from interaction environments associated with immune regulation	Reduces risk of disrupting protective or homeostatic interactions

**Table 3:** Spatial technologies for niche-aware therapeutic interpretation.

Technology Class	Main Strengths	Main Limitations	Translational Applications
Spatial transcriptomics	Broad molecular profiling with preservation of tissue location	Resolution varies across platforms, low-abundance signals may be missed, and tissue coverage can limit throughput	Mapping pathway activity to tissue regions, identifying disease-associated niches, and supporting target prioritisation
Single-cell spatial transcriptomics	High-resolution linkage between cellular state and tissue organisation	Greater technical complexity, higher cost, and limited scalability across large cohorts	Defining cellular ecosystems, resolving local immune programmes, and linking spatial organisation to therapeutic response
Multiplex imaging	Protein-level localisation with high spatial resolution and direct visualisation of cellular neighbourhoods	Limited marker number, antibody dependence, segmentation uncertainty, and image-analysis complexity	Quantifying immune localisation, cell–cell proximity, tissue compartmentalisation, and pathology-compatible biomarkers
Digital pathology and AI-assisted histology	Scalable use of routine tissue images and compatibility with clinical workflows	Requires robust training datasets, external validation, and careful biological interpretation	Inferring tissue microenvironment features, supporting patient stratification, and developing clinically deployable spatial biomarkers
Integrated spatial computational analysis	Combines spatial, single-cell, histological, and clinical data into interpretable tissue-level features	Dependent on reference quality, model assumptions, and harmonisation across platforms	Reconstructing interaction niches, prioritising candidate pathways, and interpreting response variability across patient groups

## Data Availability

No new data were created in this study. All information discussed in this review is available in the cited published literature.

## Abbreviation

The following abbreviation is used in this manuscript:

Ref	References

## References

[1] Piao W., Lee Z.L., Zapas G., Wu L., Jewell C.M., Abdi R., Bromberg J.S. (2025). Regulatory T Cell and Endothelial Cell Crosstalk. Nat. Rev. Immunol..

[2] Mirchandani A.S., Sanchez-Garcia M.A., Walmsley S.R. (2025). How Oxygenation Shapes Immune Responses: Emerging Roles for Physioxia and Pathological Hypoxia. Nat. Rev. Immunol..

[3] José C., Almeida R., Veras F.P., Paiva I.M., Schneider A.H., Silva C., Gomes G.F., Costa V.F., Manuella B., Silva S. (2024). Neutrophil Virucidal Activity Against SARS-CoV-2 Is Mediated by Neutrophil Extracellular Traps. J. Infect. Dis..

[4] Bonilha C.S., Veras F.P., de Queiroz Cunha F. (2023). NET-Targeted Therapy: Effects, Limitations, and Potential Strategies to Enhance Treatment Efficacy. Trends Pharmacol. Sci..

[5] Bedaj M., Bonilha C.S., Mcinnes I.B., Garside P., Benson R.A., Benson R.A. (2022). Tofacitinib Inhibits CD4 T Cell Polarisation to Th1 during Priming Thereby Leading to Clinical Impact in a Model of Experimental Arthritis. Clin. Exp. Rheumatol..

[6] Prendergast C.T., Benson R.A., Scales H.E., Bonilha C.S., Cole J.J., McInnes I., Brewer J.M., Garside P. (2022). Dissecting the Molecular Control of Immune Cell Accumulation in the Inflamed Joint. JCI Insight.

[7] Bonilha C.S., Veras F.P., dos Santos Ramos A., Gomes G.F., Rodrigues Lemes R.M., Arruda E., Alves-Filho J.C., Cunha T.M., Cunha F.Q. (2025). PAD4 Inhibition Impacts Immune Responses in SARS-CoV-2 Infection. Mucosal Immunol..

[8] van de Wall S., Anthony S.M., Hancox L.S., Pewe L.L., Langlois R.A., Zehn D., Badovinac V.P., Harty J.T. (2024). Dynamic Landscapes and Protective Immunity Coordinated by Influenza-Specific Lung-Resident Memory CD8+ T Cells Revealed by Intravital Imaging. Immunity.

[9] Reina-Campos M., Monell A., Ferry A., Luna V., Cheung K.P., Galletti G., Scharping N.E., Takehara K.K., Quon S., Challita P.P. (2025). Tissue-Resident Memory CD8 T Cell Diversity Is Spatiotemporally Imprinted. Nature.

[10] Gong D., Arbesfeld-Qiu J.M., Perrault E., Bae J.W., Hwang W.L. (2024). Spatial Oncology: Translating Contextual Biology to the Clinic. Cancer Cell.

[11] Kumaran G., Carroll L., Muirhead N., Bottomley M.J. (2025). How Can Spatial Transcriptomic Profiling Advance Our Understanding of Skin Diseases?. J. Investig. Dermatol..

[12] Zhang H., Patrick M.T., Zhao J., Zhai X., Liu J., Li Z., Gu Y., Welch J., Zhou X., Modlin R.L. (2025). Techniques and Analytic Workflow for Spatial Transcriptomics and Its Application to Allergy and Inflammation. J. Allergy Clin. Immunol..

[13] Castillo R.L., Sidhu I., Dolgalev I., Chu T., Prystupa A., Subudhi I., Yan D., Konieczny P., Hsieh B., Haberman R.H. (2023). Spatial Transcriptomics Stratifies Psoriatic Disease Severity by Emergent Cellular Ecosystems. Sci. Immunol..

[14] Subudhi I., Konieczny P., Prystupa A., Castillo R.L., Sze-Tu E., Xing Y., Rosenblum D., Reznikov I., Sidhu I., Loomis C. (2024). Metabolic Coordination between Skin Epithelium and Type 17 Immunity Sustains Chronic Skin Inflammation. Immunity.

[15] Schäbitz A., Hillig C., Mubarak M., Jargosch M., Farnoud A., Scala E., Kurzen N., Pilz A.C., Bhalla N., Thomas J. (2022). Spatial Transcriptomics Landscape of Lesions from Non-Communicable Inflammatory Skin Diseases. Nat. Commun..

[16] De Lima J., Boutet M.A., Bortolotti O., Chépeaux L.A., Glasson Y., Dumé A.S., Lau R., Humbert P., Allain S., Le Pluart A. (2025). Spatial Mapping of Rheumatoid Arthritis Synovial Niches Reveals a LYVE1+ Macrophage Network Associated with Response to Therapy. Ann. Rheum. Dis..

[17] Carlberg K., Korotkova M., Larsson L., Catrina A.I., Ståhl P.L., Malmström V. (2019). Exploring Inflammatory Signatures in Arthritic Joint Biopsies with Spatial Transcriptomics. Sci. Rep..

[18] Caetano A.J., Redhead Y., Karim F., Dhami P., Kannambath S., Nuamah R., Volponi A.A., Nibali L., Booth V., D’Agostino E.M. (2023). Spatially Resolved Transcriptomics Reveals Pro-Inflammatory Fibroblast Involved in Lymphocyte Recruitment through CXCL8 and CXCL10. eLife.

[19] Massimino L., Parigi T.L., Riva M., Nicolò S., Errico C., Spanò S., Mino S., Bugatti M., Frontali A., Scarfò F. (2025). Spatiotemporal Analysis of Crohn’s Disease Reveals PECAM2 Signaling at the Basis of the Inflammation-to-Fibrosis Transition. J. Crohns Colitis.

[20] Kolachala V.L., Maddipatla S.C., Murthy S., Hwang Y., Dodd A.F., Sharma G., Munasinghe S., Pelia R.S., Venkateswaran S., Anbazhagan M. (2025). Altered Inflammatory Mucosal Signatures within Their Spatial and Cellular Context during Active Ileal Crohn’s Disease. JCI Insight.

[21] Yalom L.K., Herrnreiter C.J., Bui T.M., Lockhart J., Piccolo E.B., Ren X., Wei C., Serdiukova A., Thorp E.B., Dulai P.S. (2025). Spatially Separated Epithelium-Associated and Lamina Propria Neutrophils Present Distinct Functional Identities in the Inflamed Colon Mucosa. Mucosal Immunol..

[22] Karimi E., Yu M.W., Maritan S.M., Perus L.J.M., Rezanejad M., Sorin M., Dankner M., Fallah P., Doré S., Zuo D. (2023). Single-Cell Spatial Immune Landscapes of Primary and Metastatic Brain Tumours. Nature.

[23] Liu Y., Ye S.Y., He S., Chi D.M., Wang X.Z., Wen Y.F., Ma D., Nie R.C., Xiang P., Zhou Y. (2024). Single-Cell and Spatial Transcriptome Analyses Reveal Tertiary Lymphoid Structures Linked to Tumour Progression and Immunotherapy Response in Nasopharyngeal Carcinoma. Nat. Commun..

[24] Arora R., Cao C., Kumar M., Sinha S., Chanda A., McNeil R., Samuel D., Arora R.K., Matthews T.W., Chandarana S. (2023). Spatial Transcriptomics Reveals Distinct and Conserved Tumor Core and Edge Architectures That Predict Survival and Targeted Therapy Response. Nat. Commun..

[25] Firsova A.B., Marco Salas S., Kuemmerle L.B., Abalo X.M., Sountoulidis A., Larsson L., Mahbubani K.T., Theelke J., Andrusivova Z., Alonso Galicia L. (2025). Spatial Single-Cell Atlas Reveals Regional Variations in Healthy and Diseased Human Lung. Nat. Commun..

[26] Polonsky M., Gerhardt L.M.S., Yun J., Koppitch K., Colón K.L., Amrhein H., Wold B., Zheng S., Yuan G.C., Thomson M. (2024). Spatial Transcriptomics Defines Injury Specific Microenvironments and Cellular Interactions in Kidney Regeneration and Disease. Nat. Commun..

[27] Smith D., Eichinger A., Fennell É., Xu-Monette Z.Y., Rech A., Wang J., Esteva E., Seyedian A., Yang X., Zhang M. (2025). Spatial and Single Cell Mapping of Castleman Disease Reveals Key Stromal Cell Types and Cytokine Pathways. Nat. Commun..

[28] Lütge M., Kurz L., Stanossek Y., Meili S., Cheng H.-W., De Martin A., Brandstadter J., Maillard I., Robinson M.D., Stoeckli S.J. (2025). Fibroblastic Reticular Cells Form Reactive Myeloid Cell Niches in Human Lymph Nodes. Sci. Immunol..

[29] Launonen I.M., Niemiec I., Hincapié-Otero M., Erkan E.P., Junquera A., Afenteva D., Falco M.M., Liang Z., Salko M., Chamchougia F. (2024). Chemotherapy Induces Myeloid-Driven Spatially Confined T Cell Exhaustion in Ovarian Cancer. Cancer Cell.

[30] Vannan A., Lyu R., Williams A.L., Negretti N.M., Mee E.D., Hirsh J., Hirsh S., Hadad N., Nichols D.S., Calvi C.L. (2025). Spatial Transcriptomics Identifies Molecular Niche Dysregulation Associated with Distal Lung Remodeling in Pulmonary Fibrosis. Nat. Genet..

[31] Amisaki M., Zebboudj A., Yano H., Zhang S.L., Payne G., Chandra A.K., Yu R., Guasp P., Sethna Z.M., Ohmoto A. (2025). IL-33-Activated ILC2s Induce Tertiary Lymphoid Structures in Pancreatic Cancer. Nature.

[32] Wagner M., Ealey K.N., Tetsu H., Kiniwa T., Motomura Y., Moro K., Koyasu S. (2020). Tumor-Derived Lactic Acid Contributes to the Paucity of Intratumoral ILC2s. Cell Rep..

[33] Jacquelot N., Seillet C., Wang M., Pizzolla A., Liao Y., Hediyeh-zadeh S., Grisaru-Tal S., Louis C., Huang Q., Schreuder J. (2021). Blockade of the Co-Inhibitory Molecule PD-1 Unleashes ILC2-Dependent Antitumor Immunity in Melanoma. Nat. Immunol..

[34] Alexandre Y.O., Potemkin N., Schienstock D., Duchamp B., Poch A., Christo S.N., Li S., Qin L., Beattie L., Utzschneider D.T. (2025). Splenic Fibroblastic Reticular Cells Orchestrate Dendritic Cell Maturation and Facilitate CD8+ T Cell Priming and Protective Memory. Sci. Adv..

[35] FitzPatrick M.E.B., Antanaviciute A., Dunstan M., Künnapuu K., Trzupek D., Provine N.M., Dooley K., Zhang J.Y., Irwin S.L., Garner L.C. (2025). Immune–Epithelial–Stromal Networks Define the Cellular Ecosystem of the Small Intestine in Celiac Disease. Nat. Immunol..

[36] Bonilha C.S. (2025). The Synapse Revisited: Molecular Networks of CD4 T Cell–DC Interactions. Int. Immunopharmacol..

[37] Bonilha C.S., Benson R.A., Scales H.E., Brewer J.M., Garside P. (2021). Junctional Adhesion Molecule-A on Dendritic Cells Regulates Th1 Differentiation. Immunol. Lett..

[38] O’Neill A., Zakaria N., Bull C., Egan H., Corry S.M., Leonard N.A., O’Meara C., Howard L., Walsh A., Reidy E. (2025). Stromal Cells Modulate Innate Immune Cell Phenotype and Function in Colorectal Cancer via the Sialic Acid/Siglec Axis. J. Immunother. Cancer.

[39] Niec R.E., Rudensky A.Y., Fuchs E. (2021). Inflammatory Adaptation in Barrier Tissues. Cell.

[40] Lund A.W., Wagner M., Fankhauser M., Steinskog E.S., Broggi M.A., Spranger S., Gajewski T.F., Alitalo K., Eikesdal H.P., Wiig H. (2016). Lymphatic Vessels Regulate Immune Microenvironments in Human and Murine Melanoma. J. Clin. Investig..

[41] Wagner M., Steinskog E.S., Wiig H. (2019). Blockade of Lymphangiogenesis Shapes Tumor-Promoting Adipose Tissue Inflammation. Am. J. Pathol..

[42] Fankhauser M., Broggi M.A.S., Potin L., Bordry N., Jeanbart L., Lund A.W., Da Costa E., Hauert S., Rincon-Restrepo M., Tremblay C. (2017). Tumor Lymphangiogenesis Promotes T Cell Infiltration and Potentiates Immunotherapy in Melanoma. Sci. Transl. Med..

[43] Scholaert M., Houmadi R., Martin J., Serhan N., Tauber M., Braun E., Basso L., Merle E., Descargues P., Viguier M. (2023). 3D Deconvolution of Human Skin Immune Architecture with Multiplex Annotated Tissue Imaging System. Sci. Adv..

[44] Smithmyer M.E., Hu A., Dufort M.J., Hocking A.M., Wiedeman A.E., Fasano K.J., Torgerson T.R., Skene P.J., Reading J., Li X. (2025). Longitudinally Stable T Cell Function and Innate Immune Activation Distinguish Healthy Adult Immunotypes. Sci. Transl. Med..

[45] Zotova N., Zhuravleva Y., Chereshnev V., Gusev E. (2023). Acute and Chronic Systemic Inflammation: Features and Differences in the Pathogenesis, and Integral Criteria for Verification and Differentiation. Int. J. Mol. Sci..

[46] Holst-Hansen T., Nielsen P.Y., Jensen M.H., Mandrup-Poulsen T., Trusina A. (2024). Tipping-Point Transition from Transient to Persistent Inflammation in Pancreatic Islets. npj Syst. Biol. Appl..

[47] Mennillo E., Kim Y.J., Lee G., Rusu I., Patel R.K., Dorman L.C., Flynn E., Li S., Bain J.L., Andersen C. (2024). Single-Cell and Spatial Multi-Omics Highlight Effects of Anti-Integrin Therapy across Cellular Compartments in Ulcerative Colitis. Nat. Commun..

[48] Thomas T., Friedrich M., Rich-Griffin C., Pohin M., Agarwal D., Pakpoor J., Lee C., Tandon R., Rendek A., Aschenbrenner D. (2024). A Longitudinal Single-Cell Atlas of Anti-Tumour Necrosis Factor Treatment in Inflammatory Bowel Disease. Nat. Immunol..

[49] Guo A., Ross C., Chande N., Gregor J., Ponich T., Khanna R., Sey M., Beaton M., Yan B., Kim R.B. (2022). High Oncostatin M Predicts Lack of Clinical Remission for Patients with Inflammatory Bowel Disease on Tumor Necrosis Factor α Antagonists. Sci. Rep..

[50] Rivellese F., Nerviani A., Giorli G., Warren L., Jaworska E., Bombardieri M., Lewis M.J., Humby F., Pratt A.G., Filer A. (2023). Stratification of Biological Therapies by Pathobiology in Biologic-Naive Patients with Rheumatoid Arthritis (STRAP and STRAP-EU): Two Parallel, Open-Label, Biopsy-Driven, Randomised Trials. Lancet Rheumatol..

[51] Battat R., Sangiorgi B., Linggi B., Gui X., Smith M.I., Mehandru S., Longman R., Lukin D.J., Scherl E.J., Qin L. (2025). Spatial Transcriptomics Reveals Unique Inflammatory Signatures across All Anatomic Locations in Postoperative Crohn’s Disease. J. Crohns Colitis.

[52] Wang X.Q., Danenberg E., Huang C.S., Egle D., Callari M., Bermejo B., Dugo M., Zamagni C., Thill M., Anton A. (2023). Spatial Predictors of Immunotherapy Response in Triple-Negative Breast Cancer. Nature.

[53] Yoshimoto M., Sugihara K., Tokumura K., Tsuji S., Hinoi E. (2025). Integrated Spatial and Single-Cell Transcriptomics Reveals Poor Prognostic Ligand–Receptor Pairs in Glioblastoma. Cells.

[54] Li S., Wang R., Liu S., Li S.C. (2025). Finding Spatially Variable Ligand-Receptor Interactions with Functional Support from Downstream Genes. Nat. Commun..

[55] Bonilha C.S. (2026). Platelet–Neutrophil Niches Associate with Epidermal Immune Activation and Systemic Inflammation in Psoriatic Disease. J. Mol. Med..

[56] Magalhaes G.S., Gregorio J.F., Beltrami V.A., Felix F.B., Oliveira-Campos L., Bonilha C.S., Righetti R.F., Tibério I.d.F.L.C., De Sousa F.B., Rezende B.M. (2024). A Single Dose of Angiotensin-(1–7) Resolves Eosinophilic Inflammation and Protects the Lungs from a Secondary Inflammatory Challenge. Inflamm. Res..

[57] De Araujo S., Costa V.V.R.D.M., Santos F.M., De Sousa C.D.F., Moreira T.P., Gonçalves M.R., Franciel B.F., Queiroz-Junior C.M., Perretti M., Souza D.G. (2022). Annexin A1-FPR2/ALX Signaling Axis Regulates Acute Inflammation during Chikungunya Virus Infection. Cells.

[58] Salié H., Wischer L., D’Alessio A., Godbole I., Suo Y., Otto-Mora P., Beck J., Neumann O., Stenzinger A., Schirmacher P. (2025). Spatial Single-Cell Profiling and Neighbourhood Analysis Reveal the Determinants of Immune Architecture Connected to Checkpoint Inhibitor Therapy Outcome in Hepatocellular Carcinoma. Gut.

[59] Oh J., Hoelzl J., Carlson J.C.T., Bill R., Peterson H.M., Faquin W.C., Pittet M.J., Pai S.I., Weissleder R. (2025). Spatial Analysis Identifies DC Niches as Predictors of Pembrolizumab Therapy in Head and Neck Squamous Cell Cancer. Cell Rep. Med..

[60] Quek C., Pratapa A., Bai X., Al-Eryani G., Pires da Silva I., Mayer A., Bartonicek N., Harvey K., Maher N.G., Conway J.W. (2024). Single-Cell Spatial Multiomics Reveals Tumor Microenvironment Vulnerabilities in Cancer Resistance to Immunotherapy. Cell Rep..

[61] Thomas M.F., Slowikowski K., Manakongtreecheep K., Sen P., Samanta N., Tantivit J., Nasrallah M., Zubiri L., Smith N.P., Tirard A. (2024). Single-Cell Transcriptomic Analyses Reveal Distinct Immune Cell Contributions to Epithelial Barrier Dysfunction in Checkpoint Inhibitor Colitis. Nat. Med..

[62] Gupta T., Antanaviciute A., Hyun-Jung Lee C., Ottakandathil Babu R., Aulicino A., Christoforidou Z., Siejka-Zielinska P., O’Brien-Ball C., Chen H., Fawkner-Corbett D. (2024). Tracking in Situ Checkpoint Inhibitor-Bound Target T Cells in Patients with Checkpoint-Induced Colitis. Cancer Cell.

[63] Cui X., Liu S., Song H., Xu J., Sun Y. (2025). Single-Cell and Spatial Transcriptomic Analyses Revealing Tumor Microenvironment Remodeling after Neoadjuvant Chemoimmunotherapy in Non-Small Cell Lung Cancer. Mol. Cancer.

[64] Chi H., Pepper M., Thomas P.G. (2024). Principles and Therapeutic Applications of Adaptive Immunity. Cell.

[65] Giamarellos-Bourboulis E.J., Kotsaki A., Kotsamidi I., Efthymiou A., Koutsoukou V., Ehler J., Paridou A., Frantzeskaki F., Müller M.C.A., Pickkers P. (2025). Precision Immunotherapy to Improve Sepsis Outcomes: The ImmunoSep Randomized Clinical Trial. JAMA.

[66] Davar D., Morrison R.M., Dzutsev A.K., Karunamurthy A., Chauvin J.M., Amatore F., Deutsch J.S., Das Neves R.X., Rodrigues R.R., McCulloch J.A. (2024). Neoadjuvant Vidutolimod and Nivolumab in High-Risk Resectable Melanoma: A Prospective Phase II Trial. Cancer Cell.

[67] Feng R., Spieth L., Liu L., Berghoff S., Franz J., Liu Q., Wang Z., Tiwari V., Vitale S., Frerich S. (2025). Single-Cell Spatial Transcriptomic Profiling Defines a Pathogenic Inflammatory Niche in Chronic Active Multiple Sclerosis Lesions. Immunity.

[68] Patel B.K., Raabe M.J., Lang E.R., Song Y., Lu C., Deshpande V., Nieman L.T., Aryee M.J., Chen Y.-B., Ting D.T. (2023). Spatial Transcriptomics Reveals Distinct Tissue Niches Linked with Steroid Responsiveness in Acute Gastrointestinal GVHD. Blood.

[69] Peyraud F., Guégan J.P., Rey C., Lara O., Odin O., Del Castillo M., Vanhersecke L., Coindre J.M., Clot E., Brunet M. (2025). Spatially Resolved Transcriptomics Reveal the Determinants of Primary Resistance to Immunotherapy in NSCLC with Mature Tertiary Lymphoid Structures. Cell Rep. Med..

[70] Soupir A.C., Hayes M.T., Peak T.C., Ospina O., Chakiryan N.H., Berglund A.E., Stewart P.A., Nguyen J., Segura C.M., Francis N.L. (2024). Increased Spatial Coupling of Integrin and Collagen IV in the Immunoresistant Clear-Cell Renal-Cell Carcinoma Tumor Microenvironment. Genome Biol..

[71] Chan A.S.F., Greiner J., Marschhäuser L., Brennan T.A., Perez-Feliz S., Agrawal A., Hemmer H., Sinning K., Cheung J.W.L., Iqbal Z. (2025). Spatiotemporal Dynamics of the Cardioimmune Niche during Lesion Repair. Nat. Cardiovasc. Res..

[72] Weeratunga P., Hunter B., Sergeant M., Bull J., Clelland C., Denney L., Vuppusetty C., Burgoyne R., Woo J., Hu T. (2025). Temporo-Spatial Cellular Atlas of the Regenerating Alveolar Niche in Idiopathic Pulmonary Fibrosis. Nat. Commun..

[73] Lerma-Martin C., Badia-i-Mompel P., Ramirez Flores R.O., Sekol P., Schäfer P.S.L., Riedl C.J., Hofmann A., Thäwel T., Wünnemann F., Ibarra-Arellano M.A. (2024). Cell Type Mapping Reveals Tissue Niches and Interactions in Subcortical Multiple Sclerosis Lesions. Nat. Neurosci..

[74] Benjamin K., Bhandari A., Kepple J.D., Qi R., Shang Z., Xing Y., An Y., Zhang N., Hou Y., Crockford T.L. (2024). Multiscale Topology Classifies Cells in Subcellular Spatial Transcriptomics. Nature.

[75] Chen J.H., Nieman L.T., Spurrell M., Jorgji V., Elmelech L., Richieri P., Xu K.H., Madhu R., Parikh M., Zamora I. (2024). Human Lung Cancer Harbors Spatially Organized Stem-Immunity Hubs Associated with Response to Immunotherapy. Nat. Immunol..

[76] Patkar S., Chen A., Basnet A., Bixby A., Rajendran R., Chernet R., Faso S., Kumar P.A., Desai D., El-Zammar O. (2024). Predicting the Tumor Microenvironment Composition and Immunotherapy Response in Non-Small Cell Lung Cancer from Digital Histopathology Images. npj Precis. Oncol..

[77] Abdulrahman Z., Slieker R.C., McGuire D., Welters M.J.P., Van Poelgeest M.I.E., Van Der Burg S.H. (2025). Single-Cell Spatial Transcriptomics Unravels Cell States and Ecosystems Associated with Clinical Response to Immunotherapy. J. Immunother. Cancer.

[78] Li Y., Luo Y. (2024). STdGCN: Spatial Transcriptomic Cell-Type Deconvolution Using Graph Convolutional Networks. Genome Biol..

[79] Bonilha C.S. (2026). Ligand–Receptor Hotspots in Dendritic–T Cell Niches Expose Targets in Autoimmunity. Transl. Res..

[80] Lim H.J., Wang Y., Buzdin A., Li X. (2025). A Practical Guide for Choosing an Optimal Spatial Transcriptomics Technology from Seven Major Commercially Available Options. BMC Genom..

[81] Khan M., Arslanturk S., Draghici S. (2025). A Comprehensive Review of Spatial Transcriptomics Data Alignment and Integration. Nucleic Acids Res..

[82] Si Y., Lee J.S., Jun G., Kang H.M., Lee J.H. (2025). Spatial Omics Enters the Microscopic Realm: Opportunities and Challenges. Trends Genet..

[83] Heitz M., Ma Y., Kubal S., Schiebinger G. (2025). Spatial Transcriptomics Brings New Challenges and Opportunities for Trajectory Inference. Annu. Rev. Biomed. Data Sci..

[84] Bonilha C.S. (2026). Spatial Imbalance of Innate-like T-Cell Niches Underlies Clinical Trajectories in Psoriasis. Int. J. Mol. Sci..

[85] Bonilha C.S. (2026). Immune Triad Microenvironments Define Coordinated Activation Architecture in Psoriatic Disease. Int. Immunopharmacol..

[86] Jiménez-Santos M.J., García-Martín S., Rubio-Fernández M., Gómez-López G., Al-Shahrour F. (2024). Spatial Transcriptomics in Breast Cancer Reveals Tumour Microenvironment-Driven Drug Responses and Clonal Therapeutic Heterogeneity. NAR Cancer.

[87] Wang X., Venet D., Lifrange F., Larsimont D., Rediti M., Stenbeck L., Dupont F., Rouas G., Garcia A.J., Craciun L. (2024). Spatial Transcriptomics Reveals Substantial Heterogeneity in Triple-Negative Breast Cancer with Potential Clinical Implications. Nat. Commun..

[88] Song X., Xiong A., Wu F., Li X., Wang J., Jiang T., Chen P., Zhang X., Zhao Z., Liu H. (2023). Spatial Multi-Omics Revealed the Impact of Tumor Ecosystem Heterogeneity on Immunotherapy Efficacy in Patients with Advanced Non-Small Cell Lung Cancer Treated with Bispecific Antibody. J. Immunother. Cancer.

[89] Bonilha C.S. (2026). A Spatial Transit–Retention Axis Reveals Adaptive Immune Organisation in Psoriatic Disease. Mol. Cell. Biochem..

[90] Zhou L.F., Liao H.Y., Han Y., Zhao Y. (2024). The Use of Organoids in Creating Immune Microenvironments and Treating Gynecological Tumors. J. Transl. Med..

